# *Anopheles* mosquito diversity, entomological indicators of malaria transmission and challenges of morphological identification in southwestern Ethiopia

**DOI:** 10.1186/s41182-023-00529-5

**Published:** 2023-07-14

**Authors:** Adilo Assa, Nigatu Eligo, Fekadu Massebo

**Affiliations:** Department of Biology, Arba Minch University, Arba Minch, Ethiopia

**Keywords:** *Anopheles arabiensis*, Blood meal index, Boreda district, Morphological misidentification

## Abstract

**Background:**

A number of *Anopheles* species play either a primary or secondary role in malaria transmission. This necessitates understanding the species composition, bionomics, and behaviors of malaria mosquitoes in a particular geographic area, which is relevant to design and implement tailored intervention tools. This study aimed to assess the species composition, sporozoite infection rate, and blood meal origins of malaria mosquitoes in two malaria-endemic villages of Boreda district in Gamo Zone, southwest Ethiopia.

**Methods:**

Thirty houses, 20 for Center for Disease Control and Prevention (CDC) light traps and 10 for Pyrethrum Spray Catches (PSC) were randomly selected for bimonthly mosquito collection from October 2019 to February 2020. An enzyme-linked immunosorbent assay (ELISA) was carried out to detect the blood meal origins and circumsporozoite proteins (CSPs). The entomological inoculation rate (EIR) was calculated by multiplying the sporozoite and human biting rates from PSCs. *Anopheles gambiae* complex and *An. funestus* group samples were further identified to species by the polymerase chain reaction (PCR). *Anopheles* species with some morphological similarity with *An. gambiae* complex or *An. funestus* group were tested using the primers of the two species complexes.

**Results:**

A total of 14 *Anopheles* species were documented, of which *An. demeilloni* was found to be the dominant species. *An. arabiensis* was found to be positive for *P. falciparum* CSP with the overall CSP rate of 0.53% (1/190: 95% CI 0.01–2.9). The overall estimated *P. falciparum* EIR of *An. arabiensis* from PSC was 1.5 infectious bites/person/5 months. Of the 145 freshly fed *Anopheles* mosquitoes tested for blood meal sources, 57.9% (84/145) had bovine blood meal, 15.2% (22/145) had human blood meal origin alone, and 16.5% (24/145) had a mixed blood meal origin of human and bovine. *Anopheles demeilloni* were more likely to feed on blood meals of bovine origin (102/126 = 80.9%), while *An. arabiensis* were more likely to have blood meals of human origin. Eleven samples (2.6%; 11/420) were morphologically categorized as *An. demeilloni*, but it has been identified as *An. leesoni* (the only *An. funestus* group identified in the area) by PCR, though it requires additional verification by sequencing, because different species genes may have amplified for these species specific primers. Similarly, a small number of *An. arabiensis* were morphologically identified as *An. salbaii, An. maculipalpis* and *An. fuscivenosus*.

**Conclusions and recommendations:**

In spite of the wide variety of *Anopheles* mosquito species, *An. arabiensis* dominates indoor malaria transmission, necessitating additional interventions targeting this species. In addition, increasing entomological knowledge may make morphological identification less difficult.

## Background

Malaria threatens millions of lives globally. According to the world malaria report of 2020, an estimated 229 million cases and 409 thousand deaths occurred worldwide in 2019 [1]. Although there was a reduction in the number of malaria cases in 2019, compared to 2015, no significant progress was documented [1]. A substantial increase in malaria was documented in 2020 [2] due to the COVID-19 pandemic and population increases. Africa accounted for about 94% of global cases. This region is also known for the most notorious malaria vectors [3]. Ethiopia is one of the malaria-endemic countries and contributed a substantial number of malaria cases in 2019. *Plasmodium falciparum* and *P. vivax* are the two dominant parasites in Ethiopia [2].

In Ethiopia, malaria affects millions of people, particularly in the fertile lowland areas. Most parts of the country are favorable for malaria mosquitoes and parasite transmission. Malaria prevention and control strategies began in the 1950s as pilot projects in Ethiopia [4]. Since then, vector control has been a key preventive method for malaria. Currently, insecticide-treated bed nets (ITNs) and indoor residual spraying (IRS) are the two main malaria intervention tools [5]. The effectiveness of these interventions, however, varies due to the vector species and their behavioral responses to intervention tools. There are spatial and temporary variations in the feeding and resting behaviors and the species composition of malaria mosquitoes. For example, the principal malaria vector in a particular area could be a secondary vector in the other and vice versa. This knowledge clearly indicates the importance of continuous surveillance and monitoring of malaria mosquitoes to inform control programs [6].

Entomological information, such as vector species composition, their role in parasite transmission, feeding, and resting behaviors is crucial in a vector control program. These data provide information about the intensity of malaria transmission and help in designing and implementing an appropriate intervention [6]. Moreover, information that is collected through entomological surveillance assists in understanding the effectiveness of malaria vector control interventions and the spatial and temporal changes in vector species [7]. The accurate identification of malaria mosquitoes is highly important in a vector control program. Morphological identification in this regard plays a key role in the immediate documentation of the most important malaria vectors and the implementation of control tools [8]. It is, however, more challenging in areas where several species cohabit, as well as in areas where morphologically similar species are present. A vector control program could be affected by misidentification of species in such areas.

We used PSC and CDC light trap techniques to investigate the *Anopheles* mosquito species composition, blood feeding patterns and their role in malaria transmission in two villages of Boreda district in Gamo zone, southwest Ethiopia. Molecular speciation of *An. gambiae* complex and *An. funestus* group was carried out. PCR was used to confirm those species that were morphologically closely related to either the *An. gambiae* complex or the *An. funestus* group using specific primers from the two *Anopheles* species complexes. This study also aimed to better understand the challenges posed by morphological misclassification in entomological monitoring and surveillance. 

## Materials and methods

### Description of the study area

**Fig. 1:**
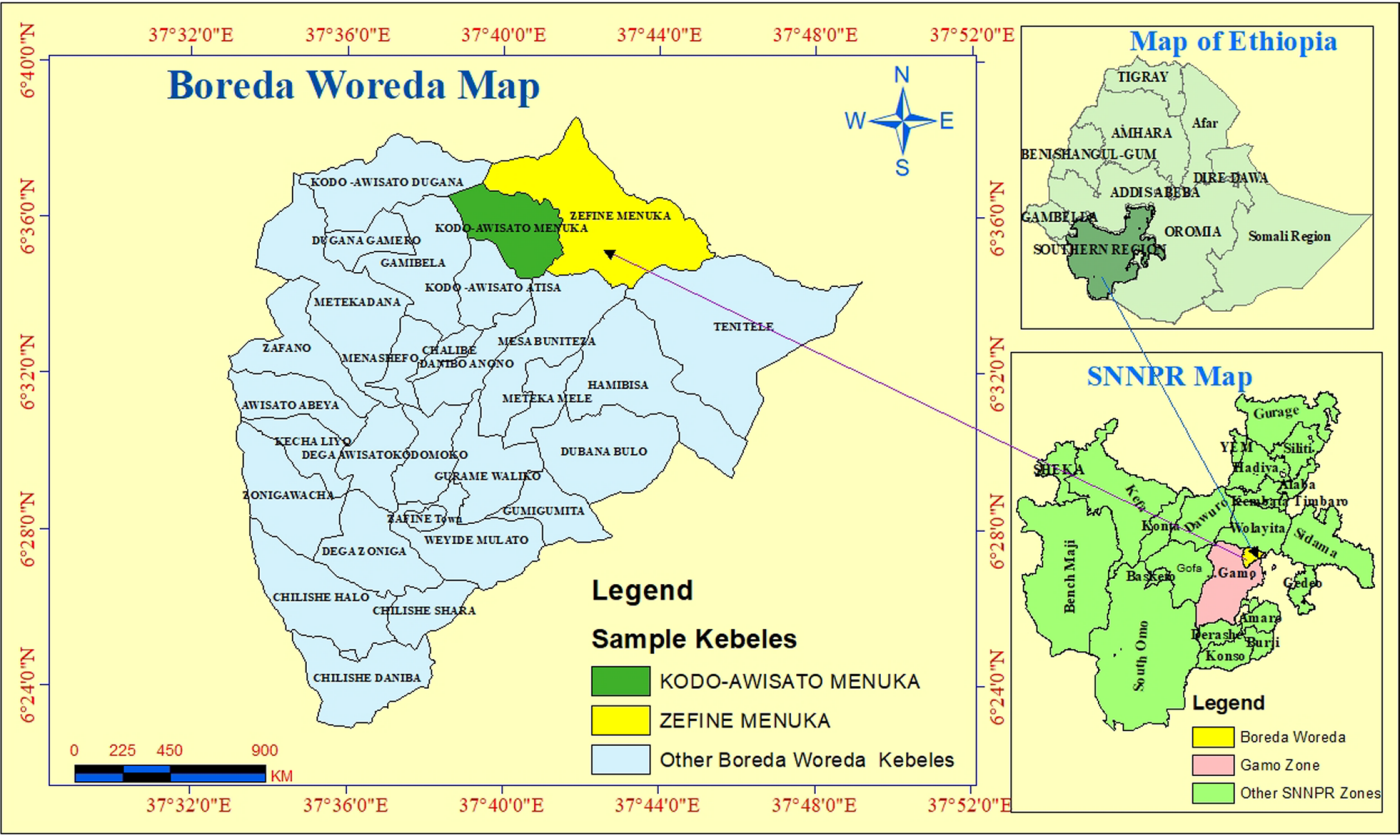
Map of the study area; SNNPR is including the new regional states

This study was conducted in the Kodo-Awsato Menuka and Zefine-Menuka *Kebeles* (= villages: the lower administrative level in Ethiopia) of Boreda district in Gamo zone (Fig. 1). Boreda district is found in Gamo zone, southwest Ethiopia. It is at a distance of 94 km from Arba Minch town, the capital of Gamo zone and is bordered with Wolaita Zone at the north, Mirab Abaya district at the south–east, Chencha district at the south–west and Kucha district at the west side of its location. The district has 31 villages and contains both highland and lowland areas. The 15 villages in the lowlands are malaria endemic. Kodo-Awsato Menuka and Zefine-Menuka villages are among the lowland villages of the district that are located about 16 km from Zefine town, the central town of the district. There were 270 households in Kodo-Awsato Menuka village and 390 in Zefine-Menuka village. The estimated population of Kodo-Awsato Menuka village is 1830 and it was 2680 in Zefine-Menuka village.

Agriculture is the basic economic activity for the inhabitants of the two villages. The villages are malarious. This study was carried out in localities with high malaria transmission to generate entomological data which provide information on the local disease transmission pattern and dynamics.

### Study design and mosquitoes sampling

Entomological sampling was done for 5 months from October 2019 to February 2020 using two entomological sampling techniques, such as pyrethrum spray and CDC light traps.

### Pyrethrum spray collection

PSC was employed to sample indoor resting adult *Anopheles* mosquitoes in selected households. Five houses were selected randomly from each study village for the indoor resting mosquito collection. The selected households were visited twice monthly. Informed consent was obtained from all subjects and/or their legal guardian(s).

The indoor resting collection was carried out in the morning from 6:00 am to 8:00 am. KWiK insecticide all-purpose insect killer with striking powder (made in Kuwait; ingredients: pyrethroid with PBO) was used for spraying the houses to knock down the indoor resting mosquitoes. The openings of the roofs and eaves were closed by a piece of cloth to prevent the mosquitoes from escaping, and the food items in the room were removed and heavy food storages closed carefully before spraying the houses. Then, the floor and furniture were covered with a white sheet and the spray administered by two persons, one outside and the other inside the house, starting at one side and moving around the house in a circular manner. The house was kept closed for 10 min and the knocked-down mosquitoes were collected from the white sheet. The mosquitoes were then transferred to vials using forceps and preserved with silica gel before traveling to Arba Minch University Medical Entomology Laboratory for further analysis.

### CDC light traps collection

Twenty houses, 10 from each village, were selected at random to collect indoor host-seeking mosquitoes using CDC light traps (Model 512, John W. Hock Company, Gainesville, Florida, USA). The CDC light traps were put in place 45 cm above the feet of a person who slept beneath an insecticide-treated mosquito net that was subsequently thoroughly cleaned. The traps were hung at 7:00 p.m. and collected at 7:00 a.m. the next morning. All other people in the trapping room were instructed to sleep under the insecticide net. The trap bags were tightened to prevent mosquitoes from escaping the bags. The mosquitoes were then transferred to vials and preserved with silica gel. The specimens were transported to Arba Minch University Medical Entomology Laboratory for further analysis.

### Mosquito identification

The standard morphological key was used to identify female *Anopheles* mosquito species [9]. Female *Anopheles* mosquitoes were categorized as unfed, fed, gravid, or half-gravid using a microscope. The sibling species of *An. gambiae* complex [10] and *An. funestus* group [11] were further identified by PCR in the Arba Minch University Medical Entomology laboratory. Some species morphologically closely related to *An. gambiae* or *An. funestus* were tested using the primers of *An. gambiae* complex and *An. funestus* group. *Anopheles gambiae* complex*, An. salbaii, An. dancalicus, An. maculipalpis* and *An. fuscivenosus* were tested using primers of *An. gambiae* complexes, whereas the *An. demeilloni* and *An. funestus* group were tested using primers of *An. funestus* complexes. Legs and wings were used for the molecular identification of the *Anopheles* species.

### CSPs detection

The female *Anopheles* mosquitoes were processed for CSPs detection in the Arba Minch University Medical Entomological laboratory. The heads and thoraces of all the collected female *Anopheles* mosquitoes were used for the detection of the CSPs of *P. falciparum* and *P. vivax*_210 malaria parasites using Enzyme-Linked Immuno-Sorbent Assay (ELISA) [12]. The heads and thoraces were homogenised in 50 μL of blocking buffer (BB) using a plastic grinder. Then, 100 μL of BB was added twice to bring the final volume to 250 μL per mosquito. The BB was removed from the plates after 1 h; 50 μL of each homogenized mosquito was added per plate. The *P. falciparum* and *P. vivax*_210 positive sample and laboratory-colony of *An. arabiensis* were used as negative controls, respectively. After 2 h incubation, the plates were washed twice with PBS-Tween 20. Then, Horseradish peroxidase (HRP)-conjugated monoclonal antibody was added to each plate, and after 1 h of incubation, the plates were washed 3 times with PBS-Tween 20. Finally, 100 μL of peroxidase substrate was added per well and incubated for 30 min. The plates were observed visually for a green color and their optical density was determined at 414 nm in the microplate ELISA reader. Samples with a green color and with optical density values of greater than two times the average optical density of the negative controls were considered as sporozoite-positive.

### Blood–meal origin assay

Freshly fed *Anopheles* mosquitoes from CDC light traps and PSC were examined for blood–meal origins determination with human and bovine antibodies by the ELISA technique [13] in different microtiter well-plates. The abdomen of *Anopheles* mosquitoes was homogenized in 50 μL of phosphate-buffered saline (PBS) solution (pH 7.4) and diluted to a volume of 200 μL by PBS. Then, 50 μL of the sample was added to each well in a 96-well microtiter plate and incubated overnight at room temperature. PBS containing a Tween-20solution was used to twice-wash each well. Then, 50 μl of host-specific conjugate (anti-human IgG and anti-bovine IgG) was added in each well of separate 96-microtiter plates and incubated for 1 h. The wells were then washed three times by PBS-Tween-20 solution and finally 100μlof peroxidase was added to each well. After 30 min the absorbance of 405 nm was recorded with an ELISA plate reader (MRX Microplate Reader, Dynex Technologies, 20,151–1683, Woonsocket, Rhode Island, USA). Human and bovine blood meals were used as a positive control, and unfed laboratory reared *An. arabiensis* was used as a negative control. To record the results as positive, the absorbance value should exceed the mean plus three times the standard deviation of four negative controls.

### Data analysis and interpretation

The data were assessed and presented based on the methods utilized in the data collection. All the data were entered and analyzed using SPSS version 20. The entomological inoculation rate and sporozoite rate were calculated. The sporozoite rate (SR) is the fraction of vector mosquitoes with *Plasmodium* sporozoite protein in their salivary glands. The entomological inoculation rate (EIR) is calculated as the product of the human-biting rate and the sporozoite rate [14]. The SR and human-biting rate (HBR) were determined for PSC catches. For PSC-based EIR, the standard EIR was calculated as (number of sporozoite-positive ELISAs/number of mosquitoes tested) × human-biting rate (HBR) (which is the number of freshly fed mosquitoes per total number of occupants in the collection room). A Chi-square test was carried out to compare the human blood index (HBI) and bovine blood index (BBI) of the *Anopheles* mosquitoes. This statistical analysis was done to assess the *Anopheles* mosquito's tendency for blood–meal sources.

### Ethical approval

Informed consent was obtained from all household heads and/or their legal guardian(s). The study was conducted in accordance with the Declaration of Helsinki and the protocol was reviewed and approved by the Ethics Review Board of Arba Minch University (CMHS/12033594). Verbal consent was obtained from village and district authorities before carrying out the mosquito collection. The objective of the study was explained to all concerned bodies.

## Results

### Anopheles species composition

A total of 680 *Anopheles* mosquitoes were collected by CDC light traps (*n* = 550; 80.9%) and PSC (*n* = 130; 19.1%). Fourteen *Anopheles* mosquito species were morphologically identified in the study area (Table 1). *Anopheles demeilloni* was the predominantly species collected (61.7%), followed by *An. arabiensis* (28.8%).

**Table 1:** Morphologically identified *Anopheles* mosquitoes species collected by CDC light traps and PSC from villages in Boreda district, southwest Ethiopia (October 2019–February 2020)

Species	Collection methods		Total	%
	CDC	PSC		
*An. demeilloni*	355	65	420	61.7
*An. gambiae* complex	148	48	196	28.8
*An. coustani*	8	1	9	1.3
*An. cinereus*	3	1	4	0.6
*An. dancalicus*	1	0	1	0.14
*An. funestus* group	4	0	4	0.6
*An. fuscivenosus*	3	0	3	0.4
*An. maculipalpis*	4	1	5	0.7
*An. salbaii*	4	2	6	0.9
*An. pretoriensis*	7	2	9	1.3
*An. rhodesiensis*	1	0	1	0.14
*An. tenebrosus*	2	0	2	0.3
*An. garnhami*	0	4	4	0.6
*An. longipalpis*	0	1	1	0.14
Unidentified	10	5	15	2.2
	550	130	680	

### Molecular identification of *Anopheles* species

A total of 635 *Anopheles* mosquitoes were tested using the primers of either *An. gambiae* or *An. funestus* complexes among those with some morphological closeness. There was morphological misidentification of *Anopheles* species. Among the 420 that were morphologically identified as *An. demeilloni* and tested for *An. funestus* complex, 11 (2.6%) were *An. leesoni*, the species of the *An. funestus* complex. Of the six that were morphologically identified as *An. salbaii* and tested for *An. gambiae* complexes, four were *An. arabiensis*. Furthermore, *An. arabiensis* were morphologically misidentified as *An. maculipalpis* and *An. fuscivenosus* (Table 2).

**Table 2:** Molecular identification of *Anopheles* mosquitoes collected from Boreda district of Gamo zone, southwest Ethiopia (October 2019–February 2020)

Morphologically identified spp.	PCR confirmed using *An. gambiae* complex primers			PCR confirmed using *An. funestus* complex primers			
	# Tested	# PCR positive	PCR identified spp.	Morphologically identified spp.	# Tested	# PCR positive	PCR identified spp.
*An. gambiae*	196	183 (93.4%)	*An. arabiensis*	*An. demeilloni*	420	11 (2.6%)	*An. leesoni*
*An. salbaii*	6	4 (66.7%)	*An. arabiensis*	*An. funestus*	4	4 (100%)	*An. leesoni*
*An. dancalicus*	1	0	None				
*An. maculipalpis*	5	2 (40%)	*An. arabiensis*				
*An. fuscivenosus*	3	1(33.3%)	*An. arabiensis*				
Total	211	190			424	15	

^#^Number

Of the 196 *An. gambiae* complex that were tested for sibling species, 183 (93.4%) were amplified for *An. arabiensis*; the rest were failed to amplify. *Anopheles leesoni* was the only *An. funestus* group identified in the area.

### Plasmodium CSP rates

A total of 680 female *Anopheles* mosquitoes (190 *An. arabiensis* and 490 other species including unidentified specimens) were tested for CSPs, of which a single *An. arabiensis* was found to be positive for *P. falciparum* CSPs from PSC collection. The overall *P. falciparum* CSP rate of *An. arabiensis* was 0.53% (1/190: 95% CI 0.01–2.9).

### Entomological inoculation rate

The EIR is obtained from the product of human-biting rate (HBR) (which is the number of freshly fed mosquitoes per total number of occupants in the room for PSC and the SR). The *P. falciparum* EIR of *An. arabiensis* was 1.5 ib/p/5 months.

### Blood meal sources

A total of 145 fresh fed *Anopheles* mosquitoes, 124 from the CDC and 21 from PSC, comprising *An. demeilloni, An. arabiensis, An. leesoni* and unidentified specimens were tested to detect their blood meal origins. Among the *Anopheles* mosquitoes that were tested for blood meal origins, 84 (57.9%; 95% CI 49.5–66.1) had bovine blood origins. Only 22 (15.2%; 95% CI 9.8–22.1) fed on human blood meal alone and about 24 (16.5%; 95% CI 10.9–23.9) had mixed blood meal origins of both human and bovine (Table 3).

**Table 3:** Blood meal origins of freshly fed *Anopheles* mosquitoes collected from Boreda district of Gamo zone, southwest Ethiopia (October 2019–February 2020)

*Anopheles* species	CDC					PSC				# Not identified
	# Tested	Human	Bovine	Mixed	# Not identified	# Tested	Human	Bovine	Mixed	
*An. demeilloni*	109	10	70	21	8	17	3	9	2	3
*An. arabiensis*	10	6	2	0	2	4	3	0	0	1
*An. leesoni*	4	0	3	0	1	0	0	0	0	0
Unidentified specimens	1	0	0	1	0	0	0	0	0	0
Total	124	16	75	22	11	21	6	9	2	4

^#^Number

*Anopheles demeilloni* predominantly fed on the bovine blood meal origins and showed statistical significance (Table 4). The bovine blood index (BBI) of *An. demeilloni*, including the mixed blood meal origins was 80.9 (95% CI 73.0–87.4); the human blood index (HBI), including mixed blood meals was 28.6 (95% CI 20.9–37.3). *Anopheles arabiensis* had blood meal sources of both human and bovine, though most of them had more human blood origins than bovine which was also statistically significant (Table 4). The HBI of *An. arabiensis* was 64.3% (95% CI 35.1–87.2), while the BBI was 14.3% (95% CI 1.8–42.8).

**Table 4:** Comparison of human (including mixed) and bovine blood meal origins (including mixed) of freshly fed *An*. *demeilloni* and *An. arabiensis* collected from Boreda district of Gamo zone, southwest Ethiopia (October 2019–February 2020)

		χ^2^ Value	*p* value (2-tail)	Odds ratio (95% CI)
# *An. demeilloni* tested	126			
Human	36			Ref.
Bovine	102	60.7	< 0.0001	7.2 (4.4–12.3)
Negative	11			
# *An. arabiensis* tested	14			
Human	9	5.4	0.001	9.7 (1.6–86.9)
Bovine	2			Ref.
Negative	3			

^#^Number

## Discussion

About 14 *Anopheles* species were morphologically identified in the Boreda district of southwest Ethiopia. Though *An. demeilloni* was the dominant anopheline species, only *An. arabiensis* was found to be positive for *P. falciparum* CSPs, demonstrating that *An. arabiensis* continues to be an important vector of malaria in this region. Furthermore, the blood meal source analysis demonstrated that *An. demeilloni* prefers to feed on bovine sources, while *An. arabiensis* were more likely to have blood meals of human origin. There was morphologically misidentification of *An. leesoni* from *An. funestus* group and *An. arabiensis* from *An. gambiae* complex*.*

There was high species diversity of malaria mosquitoes in the study district. Unlike many other studies in the region [15–17], *An. demeilloni* was the dominant species in the current study villages. Though the dominance of *An. arabiensis* varied, its principal role in malaria transmission was the same as is documented in many other studies. ITNs and IRS have brought change in the species composition and dynamics of malaria mosquitoes in many malaria-endemic countries [18]; little or no change has been documented against *An. arabiensis*. After the long implementation of vector control tools, *An. arabiensis* has maintained its position as a primary vector of malaria in many malaria endemic countries [18]. Control of this species may demand more intervention tools in addition to the existing ones [19]. There might be an increased level of malaria transmission outdoors in the present day due to the changing behavior of malaria mosquitoes. The need to collect malaria mosquitoes outdoors is crucial to assess the risk of people working at night outdoors. Moreover, the predominance of *An. demeilloni* and the observed human–vector contact may require attention for further research to comprehend the role of this species.

From the total tested *Anopheles* mosquitoes for CSPs, *An. arabiensis* was found to be positive for *P. falciparum* CSP. The overall *P. falciparum* CSP rate of *An. arabiensis* was comparable with the CSP rate from Sille village in 2006 [15] and Chano Mille in 2013 [17]. However, there is a methodological difference; in the current study, entomological sampling was carried out by CDC light traps and PSC, while Taye et al. [15] was carried out by HLC. The collection method that was employed by Massebo et al. [17] was similar to the methods used in the current study. The findings of the current and previous studies have shown that *An. arabiensis* continues to play a primary role in malaria transmission in Ethiopia [16, 17, 20]. On the other hand, it is important to understand the variation in the CSP rate of *An. arabiensis* in different areas, and the method of collection [20]. We did not confirm the presence of the *Plasmodium* parasite by a PCR, nor did we expose it to heat to check for the heat stability of the antigen. Therefore, there could be the possibility of a false positive.

The EIR of *An. arabiensis* was calculated from the PSC collection. Accordingly, the *P. falciparum* EIR of *An. arabiensis* was 1.5ib/p/5 months, indicating the existence of active malaria transmission. There are a few studies who have attempted to estimate the EIR in Ethiopia by following similar mosquito sampling techniques [17, 21]. Therefore, it could be possible to compare the current result with other studies. This result is comparable with a study in south-central Ethiopia [21] and the southwest Rift Valley [17], and it implies that this species plays a key role in malaria transmission in the region. Future malaria control programs might focus on expanding the control toolbox to address the challenge that is related to *An. arabiensis* [22]. This species has opportunistic and flexible feeding and resting behaviors which could challenge future control programs [23].

The blood meal origins test result of the current study showed that most of *An. demeilloni* displayed the tendency to feed on bovine blood meal, while *An. arabiensis* showed the tendency to feed on human blood meal. Similar studies confirmed the tendency of *An. demeilloni* to feed on bovine blood meal [24]. In the current study, *An. arabiensis* exhibited a tendency to feed on human blood rather than bovine blood, somewhat contrasting other studies that documented the bovine blood feeding tendency of *An. arabiensis* [24, 25]. Several studies have indicated the opportunistic feeding behavior of *An. arabiensis* which is mainly determined by the accessibility of the hosts [26, 27].

There was a morphological misidentification of *Anopheles* mosquitoes. Species with some morphologically similar structures with *An. gambiae* or *An. funestus* complexes were molecularly identified using the primers of the two species. Among the *An. salbaii, An. dancalicus, An. maculipalpis* and *An. fuscivenosus* that were morphologically identified, a substantial number were *An. arabiensis*. Similarly, a substantial number of *An. leesoni* were morphologically identified as *An. demeilloni*. This implies that morphological identification alone may result in the wrong speciation of *Anopheles* mosquitoes due to the morphological similarity of some species, and this in turn might affect malaria control and elimination programs. Furthermore, morphological identification alone might contribute to the wrong documentation of *Anopheles* species. Though the molecular technique is more expensive and time consuming than a morphological technique, the correct identification of the species using molecular technique might benefit the control program [28]. Morphological misidentification of the species may occur when specimens have lost important external characteristics [8]. The knowledge of the entomology staff also has an impact on the morphological identification of the species. Thus, it is essential to advance entomological knowledge to minimize problems with misidentification.

This study has some drawbacks. Although PCR technology was used to correct the incorrect species identification of *Anopheles*, further sequencing utilising species-specific areas, such as ITS 2, is still necessary to validate the gene alignments, since various species may have genes amplified by species-specific primers. In addition, just a small number of *Anopheles* mosquitoes were examined for the source of their blood meals, and the statistical significance that resulted from this limited sample size might result in incorrect conclusions.

## Conclusions and recommendations

The current study revealed the existence of the varieties of *Anopheles* species that were identified using morphological and molecular methods. The dominant *Anopheles* species was *An. demeilloni,* followed by *An. arabiensis. Anopheles arabiensis* was the only vector of malaria in the area that was found to be positive for *P. falciparum* CSPs. According to the current blood meal sources analysis, most of the tested *Anopheles* mosquitoes displayed the tendency to feed on bovine blood meal origin, and hence animal-based intervention could be recommended. Because the *Anopheles* species was incorrectly identified based on morphology, the correct identification of the *Anopheles* species may necessitate the use of molecular techniques.

## Data Availability

All available data are included in this manuscript.

## Abbreviations

BBI: Bovine blood index
CDC: Center for disease control and prevention
CSPs: Circumsporozoite proteins
EIR: Entomological inoculation rate
ELISA: Enzyme-linked immunosorbent assay
HBI: Human blood index
HBR: Human biting rate
HLC: Human landing catches
IRS: Indoor residual spray
ITNs: Insecticide treated nets
MOH: Ministry of Health
PCR: Polymerase chain reaction
PSC: Pyrethrum spray catches
RDT: Rapid diagnostic tests
WHO: World health organization
SR: Sporozoite rate
PBO: Pyrethroid-piperonyl butoxide
PBS: Phosphate buffered saline
DNA: Deoxyribonucleic acid
